# Current techniques for visualizing RNA in cells

**DOI:** 10.12688/f1000research.8151.1

**Published:** 2016-04-28

**Authors:** Lilith V.J.C. Mannack, Sebastian Eising, Andrea Rentmeister

**Affiliations:** 1 Department of Chemistry, Institute of Biochemistry, University of Münster, Münster, Germany; 2 Cells in Motion, Cluster of Excellence, Münster, Germany

**Keywords:** RNA, visualization, imaging, proteins

## Abstract

Labeling RNA is of utmost interest, particularly in living cells, and thus RNA imaging is an emerging field. There are numerous methods relying on different concepts ranging from hybridization-based probes, over RNA-binding proteins to chemo-enzymatic modification of RNA. These methods have different benefits and limitations. This review aims to outline the current state-of-the-art techniques and point out their benefits and limitations.

## Introduction

The localization of mRNA in the cell has been a topic of interest since the 1980s, when protein localization was linked to localized mRNA translation ^1^. At that time, the only method by which RNA could be visualized was *in situ* hybridization (ISH) ^2^. Since then, the options for RNA detection have expanded greatly. Labeling RNA—particularly mRNA—is of utmost interest, as mRNA localization has been shown to be important in a range of situations. For example, in a developing *Drosophila* oocyte, asymmetrically localized mRNA produces a *bicoid* protein gradient through localized translation, which specifies the anterior-posterior polarity of the developing larva ^3^. In neurons, localization of mRNA is also particularly important, and localized mRNA leads to multiple rounds of translation at the synapse and activity-dependent changes ^4^. Additionally, since the number of genes transcribed was found to surpass the amount of protein-coding genes, interest in non-coding RNAs has increased ^5^. Thus, imaging microRNAs or small interfering RNAs (siRNAs) is also of interest ^6^. Furthermore, defects in mRNA localization play an important role in some diseases such as fragile X syndrome ^7^, and non-coding RNAs have been shown to be important in diseases such as cancers ^8^.

## Hybridization methods

**Figure 1.**
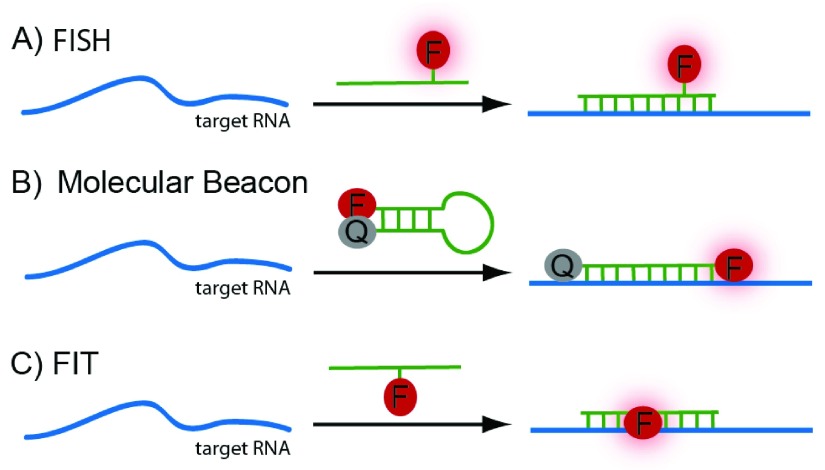
Hybridization-based methods for RNA imaging. ( **A**) Standard fluorescence *in situ* hybridization (FISH): a fluorophore-linked RNA probe binds the target RNA sequence. ( **B**) Molecular beacon: signal to noise is improved relative to a standard FISH probe because the fluorescence signal of the reporter probe is quenched when unbound. ( **C**) Forced intercalation (FIT) probes: binding enforces intercalation of the dye molecule into the probe-target duplex, resulting in a strong turn-on effect of the fluorophore.

ISH is used to visualize RNA containing a known sequence. A DNA or RNA strand of complementary sequence hybridizes to the RNA strand of interest via Watson-Crick base pairing. The probe bears features that enable its visualization (e.g., a fluorophore; Figure 1A). Fluorescence *in situ* hybridization (FISH) can distinguish between RNA molecules that differ in only a single base ^9^. FISH is highly sequence-specific, and individual RNA strands may be detected when combined with various amplification procedures in fixed cells ^9, 10^. A variety of derivatives of ISH that reduce background signal have been developed, most notably molecular beacons ( Figure 1B). Molecular beacons consist of a DNA probe that is linked to a fluorophore at one end and a quencher at the opposite end. When unbound, the probe folds into a hairpin structure, bringing the fluorophore and quencher together, thereby inhibiting fluorescence. Upon target recognition, the probe anneals and stretches out, separating the quencher from the fluorophore and enabling fluorescence ^11^. Molecular beacons have been advanced further since the 1990s. For example, the types of quencher used have been expanded to include nanoparticles ^12^. Microinjected molecular beacons can mislocalize to the nucleus in live cells; however, incorporating a tRNA sequence was shown to abrogate this problem ^13^.

An alternative approach to increase the specific fluorescent signal upon binding is to use forced intercalation (FIT) probes ( Figure 1C). FIT probes are peptide nucleic acid (PNA) or DNA single strands containing a base surrogate (typically, thiazole orange) that intercalates between the Watson-Crick base pairs and fluoresces only upon exact hybridization ^14, 15^. Their strong turn-on effect (~30-fold) makes FIT probes an attractive improvement of ISH. FIT probes have been expanded to contain dyes that emit in the blue and green ranges ^16, 17^ and have been successfully used for mRNA visualization in cells and *Drosophila* embryos ^14, 18^.

Hybridization-based RNA detection is an excellent tool for use in fixed samples and can be used in living cells and organisms when strong turn-on effects are achieved (e.g., molecular beacons and FIT probes). However, probes based on modified nucleic acids or derivatives thereof are neither cell-permeable nor can they be produced by the cell itself. Furthermore, hybridization has to occur in regions of the target RNA free of secondary structure, and hybridization conditions are typically not optimized for the cellular milieu. Recently, probes and conditions have started to be developed for use in live cells; this approach is termed fluorescence *in vivo* hybridization (FIVH) ^19– 21^. In particular, 2′- *O*-methylated oligonucleotides exhibit faster hybridization kinetics, increased melting temperatures, enhanced binding specificity, improved nuclease stability, and the ability to bind structured molecules—properties beneficial for FIVH probes ^20^. They were used to detect a variety of RNA types, such as snRNAs, rRNA, and poly(A) RNA ^20^. Nevertheless, these probes need to be introduced into the cells and thus FIVH requires transient permeabilization of cells.

## Aptamers

**Figure 2.**
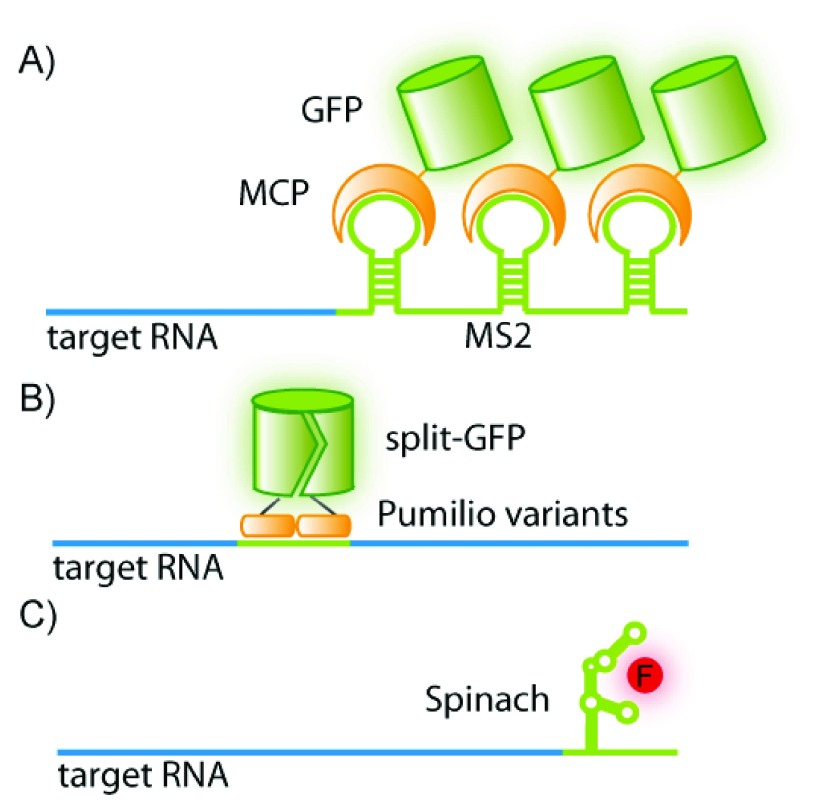
Visualization based on reporter molecules binding to a specific RNA sequence. ( **A**) A green fluorescent protein-fused-MS2 coat protein (GFP-MCP) binds a consensus sequence (MS2) appended to the RNA of interest. ( **B**) Two pumilio variants fused to different halves of split-GFP recognize a target sequence within an RNA molecule of interest. ( **C**) The aptamer “Spinach” folds to bind a turn-on fluorophore and can be appended to an RNA of interest.

RNA aptamers are another form of nucleotide-based probe but work on a different principle to the above mentioned hybridization probes. Aptamers are short, single-stranded oligonucleotides capable of binding specific target molecules based on their shape and can be obtained by *in vitro* selection ^22^. Recently, an aptamer termed “Spinach” was selected that folds to allow binding of a small-molecule fluorophore that fluoresces only upon binding the RNA aptamer ( Figure 2C) ^23^. Herein, the reporter and probe are contained within the same oligonucleotide. The aptamer sequence can be appended to the RNA of interest to enable visualization of that RNA upon binding of the fluorophore ^23^. RNA aptamers in conjunction with said small fluorophore are available in a range of colors from blue to red ^23^ and have been improved to further enhance binding efficiency and fluorescence strength ^24^. Additionally, the folding properties have been optimized for the cellular milieu ^25, 26^. A downside of RNA aptamers is the potential impediment in localization or function of some RNAs by the “Spinach” RNA tag.

## Particle-associated hybridization-based imaging probes

The group of Mirkin describe a nanoparticle conjugated spherical nucleic acid that recognizes specific RNA targets and is capable of entering the cell without the need for transfection ^27, 28^. However, there is considerable controversy surrounding this study, mainly concerning whether these sticky- or nano-flares mark specific RNAs or merely remain in endosomes after uptake by the cell ^29^. Gold nanoparticles bound to quantum dots via hybridizing DNA strands have been developed to detect specific microRNA ^30^. The microRNA triggers the dissociation of the quantum dots from the gold particle, resulting in the abrogation of quenching and thus a signal. These gold nanoparticle-quantum dot-probes bind target RNA quantitatively *in vitro*, and cell lines expressing a certain microRNA can be distinguished from cell lines that do not.

## Covalent modification of RNA in cells

**Figure 3.**
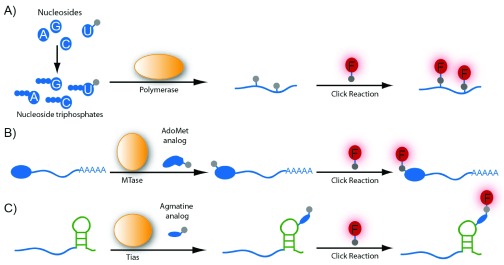
Introducing covalent modifications into RNA and subsequent labeling by click chemistry allow visualization. ( **A**) Incorporation of modified nucleotides into nascent RNA by endogenous RNA polymerases. Ethynyluridine is cell-permeable, and the respective triphosphate is made inside the cell; hence, feeding the cell with the nucleoside precursor is possible. In other examples (azido-U), the cells have to be transfected with the respective triphosphates. ( **B**) Hallmarks of RNA subtypes, such as the 5′ cap, can be selectively modified. A methyltransferase (MTase) variant can be used to modify the mRNA cap with the clickable group if the respective *S*-adenosylmethionine (AdoMet) analog is provided. ( **C**) Transcript-specific installation of a click reactive moiety can be achieved by appending a tRNA-mimicking sequence to the RNA of interest. The enzyme tRNA ^Ile2^-agmatidine synthetase (Tias) modifies the tag with a clickable group if appropriate agmatine analogs are provided.

An alternative method to mark RNA is to incorporate visualizable moieties directly into the RNA. A convenient way to achieve marking RNA without introducing a large moiety that may interfere with RNA function is to incorporate a small chemical group that may be further reacted by using click chemistry to attach to a fluorophore. There are a number of different click reactions, the most prominent being the copper(I)-catalyzed azide alkyne cycloaddition (CuAAC). Here, an azide reacts with an alkyne in the presence of Cu(I) as a catalyst. CuAAC is rapid and extremely selective; however, Cu(I) at millimolar concentrations is toxic to cells and thus this approach is limited to fixed cell samples. Jao and Salic succeeded in incorporating ethynyl groups into total RNA by feeding cells with the uridine analog 5-ethynyluridine (EU), which is converted to the respective triphosphate inside the cell ( Figure 3A) ^31^. Using a similar approach—feeding cells with *N* ^6^-propargyl adenosine—the poly(A) dynamics of mRNA could be monitored ^32^.

There are also a number of copper-free click reactions, which are more suitable for live-cell imaging, termed bioorthogonal click reactions (reviewed in 33). Sawant *et al*. synthesized an azido-modified UTP analog that can be used in the bioorthogonal strain-promoted azide-alkyne cycloaddition (SPAAC) ^34^. This allowed the click reaction to proceed in live cells; however, this UTP analog had to be transfected into the cells as its uridine precursor was no longer cell-permeable or was not a good substrate for the ribonucleoside salvage pathway ( Figure 3A).

A downside of incorporating modified nucleotides during transcription or poly(A) tail addition is that different subtypes of RNA cannot be distinguished. A possible method by which to obtain specific labeling of different subtypes is to attach chemical groups used for click reactions post-synthetically by using RNA-modifying enzymes. Subtypes of RNA may also be labeled by taking advantage of certain structures or modifications in an RNA type. For example, the 5′ cap of mRNA may be specifically labeled by using an engineered methyltransferase that is only active on the mRNA cap ( Figure 3B) ^35– 37^. This approach should be suitable in live cells because the *S*-adenosylmethionine (AdoMet) analog can be made from cell-permeable and stable methionine analogs by a variant of the methionine adenosyltransferase (MAT), which is responsible for AdoMet synthesis ^37^.

Sequence-specific RNA-modification with a propargyl group and subsequent labeling with a fluorophore have been achieved *in vitro* by using a box C/D methyltransferase-guide RNA complex and the respective propargyl-bearing analog of the cosubstrate AdoMet ^38^. Li *et al.* developed an RNA labeling system in which an RNA of interest was extended by a tRNA-derived sequence and an enzyme that specifically modifies this sequence (tRNA ^Ile2^-agmatidine synthetase, or Tias) was introduced into a cell ( Figure 3C) ^39^. This RNA-Tias combination can also accept agmatine analogs that are click-reactive and thus can be used to label RNA in cells ^39^. Similarly, a tRNA-derived recognition motif may be specifically marked by using an engineered transglycosylase that is able to transfer large visualizable groups ^40^.

## RNA-binding proteins

A number of bacteriophage-derived RNA-binding proteins have been used to mark RNA in cells. The most notable of these is the MS2-MS2 coat protein (MS2-MCP) system ( Figure 2A). This comprises a green fluorescent protein (GFP)-fused version of the bacteriophage MCP (an RNA-binding protein that recognizes a specific RNA sequence-determined hairpin) and the RNA of interest extended by multiple MS2-binding sites (MBS) ^41^. Recently, the MS2 system has been used to image single mRNA molecules in living mouse cells ^42^. This study displays both the power and drawback of this method. On the one hand, the MS2 system allows tracking and resolution of single mRNA molecules; on the other, producing a transgenic organism is very time-consuming. Another drawback is that the size of the MS2-fusion tag and the appendages to the RNA might interfere with normal mRNA function or localization ^43, 44^. Furthermore, the quantity of MS2 used must be sufficient to saturate the target RNA without raising background fluorescence, which may be difficult to achieve ^45^.

Another RNA-binding protein worth mentioning is pumilio. Pumilio is a member of the RNA-binding protein family PUF ^46^. Like many RNA-binding proteins, pumilio is modularly composed of domains that can be engineered to alter the specific RNA sequence bound ^47– 49^. Pumilio is of particular interest as it can target RNA directly without the need to introduce an RNA tag into the target RNA.

An advantage of using RNA-binding proteins to visualize RNA is that two individual RNA sequences may be targeted by separate RNA-binding proteins, thus allowing the imaging of the association of two RNA molecules of interest ^50, 51^. The potential drawback of high background fluorescence due to unbound protein may be countered by using a split GFP, which fluoresces only upon dimerization ( Figure 2B) ^52, 53^.

## Reporter protein expression by trans-splicing to visualize RNA

Two approaches have been developed by which a pre-mRNA may be spliced into a functional form, which allows the expression of a reporter protein. This enables tissue-specific localization of an mRNA of interest, although the resolution at a subcellular level is lost. Bhaumik *et al.* described a method based on trans-splicing that results in the expression of luciferase in cells of a living organism microinjected with an exogenous RNA that was processed to pre-mRNA ^54^. So *et al.*, employing a similar approach, developed an engineered ribozyme, which fuses a reporter gene to a specific gene of interest ^55^. The authors were able to detect p53 in a whole organism and on a cellular level. Despite theoretical expansion potential ^56^, the approach taken by the Gambhir lab has not been significantly developed since the publication of the original study, leaving it with the limitation that only exogenous RNA can be visualized. Similarly, the work of So *et al*. has not been further developed.

## Conclusions: current applications and outlook

Imaging of RNA is of interest at the level of both single cells and the whole organism. Labeling RNA in a single cell can show the localization of a specific transcript, which may have important biological consequences ^57^. RNA imaging at the whole organism level is important to determine the tissue expression pattern of a specific transcript. RNA labeling has seen extensive use in imaging of infection by RNA viruses (e.g., 58). Another interesting application of RNA imaging has been to monitor transcription and this has been used, for example, to determine the toxicity of certain substances that inhibit transcription ^59^.

In summary, RNA may be visualized by a variety of methods. RNA may be seen via hybridization of a reporter molecule, most commonly through FISH or variations thereof. Alternatively, RNA-binding proteins that bind specific sequences may mark an RNA molecule of interest, or an RNA aptamer that fluoresces upon binding of a fluorophore may be incorporated into the target molecule. RNA may be sequence- or subtype-specifically labeled by using click chemistry. Challenges facing the field of RNA imaging are the cell permeability of dyes used and the low abundance of target RNA. Furthermore, no method of RNA labeling is yet able to yield quantitative data on its target RNA. However, with continued development, RNA imaging will continue to provide important biological insights.
